# Trends and spatial distribution of pulmonary tuberculosis in China: a surveillance study

**DOI:** 10.3389/fpubh.2026.1866155

**Published:** 2026-07-01

**Authors:** Mengdi Chen, Tao Ding, Wenping Li, Yonghao Li, Siqi Xiong, Lulu Zhang

**Affiliations:** Department of Health Services, Naval Medical University, Shanghai, China

**Keywords:** China, forecasting, prophet, pulmonary tuberculosis, SARIMA, spatio-temporal analysis, XGBoost

## Abstract

**Background:**

Tuberculosis (TB) remains a leading infectious cause of death worldwide, and China remains a high-burden country. Pulmonary tuberculosis (PTB) accounts for most TB cases in China, but previous studies have been geographically limited, used short forecasting horizons, or lacked systematic model comparisons. This study aimed to assess trends, spatial dynamics, and future trajectories of PTB incidence and mortality across mainland China and identify geographical anomalies requiring targeted interventions.

**Methods:**

Monthly PTB incidence and mortality data for 2004–2025 were obtained from the National Notifiable Disease Reporting System. Joinpoint regression analyzed temporal trends (2004–2023). Global/local Moran’s I examined spatial clustering. A rolling-window framework (training: 2004–2021; test: 2022–2025) compared Holt-Winters, SARIMA, Prophet, and XGBoost models, forecasting to 2030. Performance was assessed using MAE, RMSE, MAPE, SMAPE, and the Diebold-Mariano test.

**Results:**

During 2004–2023, average annual PTB incidence was 66.23/100,000, and mortality was 0.20/100,000. Incidence peaked in 2005 and then declined significantly from 2006 (APC = −4.37, 95% CI: −7.60 to −3.86%). Mortality also decreased (APC = −2.43, 95% CI: −4.12 to −0.71%). Spatial hotspots shifted from southern China (2004–2008) to western China (Tibet, Xinjiang) after 2008. Liaoning Province exhibited a unique upward mortality trend (AAPC = 7.89%, *p* < 0.001) despite being a persistent cold spot. XGBoost achieved the lowest forecast errors for both incidence (MAE = 0.49, MAPE = 12.72%) and mortality (MAE = 0.0050, MAPE = 23.22%). XGBoost projects that incidence will plateau at approximately 3.5–4.2 per 100,000 through 2030, rather than continuing its historical decline; mortality is expected to remain stable at a very low level (≈0.017 per 100,000).

**Conclusion:**

PTB incidence and mortality in China have declined substantially, but marked geographic disparities persist. Western provinces remain high-burden hotspots, and Liaoning’s rising mortality despite low incidence warrants urgent investigation. XGBoost provided the most accurate forecasts, though traditional models also performed reasonably. Geographically tailored interventions, sustained surveillance, and further research into regional anomalies are essential to accelerate progress toward China’s 2030 TB elimination targets.

## 1 Introduction

Tuberculosis (TB) remains one of the leading causes of death from a single infectious agent worldwide. According to the World Health Organization (WHO) Global Tuberculosis Report 2025, an estimated 10.7 million people fell ill with TB in 2024, and 1.23 million died from the disease (1).

China is among the 30 high TB burden countries, ranking fourth after India, Indonesia, and the Philippines, accounting for 6.5% of global TB cases in 2024 (1). According to the Global Burden of Disease (GBD) Study 2023, the age-standardized mortality rate of tuberculosis in China has declined substantially over the past three decades, mirroring global trends that saw tuberculosis fall from the 7th leading cause of death worldwide in 1990 to the 15th in 2023 (2). The same GBD 2023 analysis further estimated that the global age-standardized disability-adjusted life-year (DALY) rate for tuberculosis decreased by 42.2% between 2010 and 2023, reflecting sustained progress in reducing the disease burden (3). However, pulmonary tuberculosis (PTB), which accounts for the vast majority of TB cases in China, remains a notifiable Class B infectious disease with distinct epidemiological characteristics. Understanding its long-term temporal trends, spatial dynamics, and future trajectories is essential for optimizing resource allocation and guiding targeted interventions.

While previous studies have extensively documented the epidemiological characteristics of PTB in China, most have been limited in geographic scope (focusing on single provinces or cities such as Qinghai, Xinjiang, Shandong, Henan, or Inner Mongolia) or have used short forecasting horizons (typically 12 months or less) without systematic model comparison or rolling-window validation (4–17). Nationwide analyses have either been restricted to mortality trends only (15, 18), lacked spatial clustering analysis (7), or did not extend beyond 2016 (9). Furthermore, no study has comprehensively integrated long-term (2004–2025) national and provincial monthly data to simultaneously describe temporal trends (via Joinpoint regression), spatial dynamics (via global/local Moran’s I), and future trajectories using multiple forecasting models (SARIMA, Prophet, Holt-Winters, and the machine learning model XGBoost) with a rolling-window validation framework for both PTB incidence and mortality across all 31 provinces of mainland China.

To address these gaps, the present study aimed to (1) quantify the temporal trends of PTB incidence and mortality in China from 2004 to 2023 using Joinpoint regression; (2) characterize the spatial distribution and local clustering patterns of PTB incidence across 31 provinces, with a focus on hot spot shifts over time; (3) identify geographical anomalies that may require tailored interventions; and (4) evaluate and compare the forecasting performance of four models (Holt-Winters, SARIMA, Prophet, and XGBoost) for PTB incidence and mortality up to 2030, using a rolling-window framework. All data used in this study originated from the National Notifiable Disease Reporting System, ensuring consistency and reliability. The findings are intended to inform evidence-based resource allocation and policy decisions toward achieving China’s 2030 TB elimination targets.

## 2 Materials and methods

### 2.1 Data sources

Pulmonary tuberculosis (PTB) is a statutory Class B notifiable infectious disease in China. All PTB data used in this study originated from the National Notifiable Disease Reporting System (NNDRS) managed by the Chinese Center for Disease Control and Prevention (China CDC). Because different dissemination platforms publish NNDRS data with varying temporal coverage and geographic resolution, we retrieved data from three publicly accessible sources. Importantly, all sources employ the same case definitions, reporting procedures, and statistical standards as mandated by the NNDRS. (1) Monthly PTB incidence and mortality data at both national and provincial levels from January 2004 to December 2020 were extracted from the China Public Health Science Data Center.^1^ This platform ceased releasing NNDRS data after 2020. (2) Monthly PTB incidence and mortality data from January 2021 to December 2023 at both national and provincial levels, as well as national-level monthly data from January 2024 to December 2025, were obtained from two government sources. Data for 2021–2023 were collected from the National Health Commission, China (NHC).^2^ Data for 2024–2025 were collected from the National Disease Control and Prevention Administration, China (NDCPA),^3^ which has published NNDRS data since 2024. Provincial-level data after 2023 are not publicly available; therefore, all provincial analyses were restricted to 2004–2023, while national-level data for 2004–2025 were used for forecasting.

Administrative boundary data of China were obtained from the National Geomatics Center of China (NGCC).^4^ All spatial analyses and derived maps were generated using the unmodified standard base map provided by the NGCC (map review number: GS(2024)0650).

In this study, “PTB” refers specifically to pulmonary tuberculosis, which is the focus of all analyses. When discussing general tuberculosis control policies, global targets, or comparisons with the literature, the term “TB” is used interchangeably with “PTB” for brevity and consistency with international conventions.

### 2.2 Temporal analysis

This study quantitatively analyzed the temporal trends of pulmonary tuberculosis incidence and mortality from 2004 to 2023 using the Joinpoint regression model (Joinpoint Regression Program v5.2.0, National Cancer Institute, United States). The Joinpoint method partitions the observation period into contiguous segments delimited by statistically significant joinpoints, fitting a log-linear function within each segment. Model selection was performed using the weighted Bayesian Information Criterion (BIC) (19), and the optimal number of joinpoints was determined automatically with a maximum of three joinpoints (default for 20 data points) (20). Temporal trends were quantified using the annual percent change (APC) for each segment and the average annual percent change (AAPC) over the entire study period (21). The AAPC was calculated as the weighted average of the segmental slopes (weights equal to the number of years in each segment). Ninety-five percent confidence intervals for AAPC were estimated using the empirical quantile method (10,000 resamples).

### 2.3 Autocorrelation analysis

Spatial autocorrelation analysis was used to evaluate the presence and quantify the degree of spatial dependence in the distribution of a given attribute (22). In this study, we employed ArcGIS 10.8 to conduct global and local autocorrelation analyses of the spatial distribution of pulmonary tuberculosis incidence in China from 2004 to 2023, aiming to identify geographic clustering patterns. For global spatial autocorrelation, we used the Moran’s I statistic, which ranges from −1 to +1. A positive value close to +1 indicates a high degree of clustering, whereas a negative value close to −1 suggests a dispersed pattern. A value of zero implies a random spatial distribution. The significance of the estimated Moran’s I was assessed by calculating Z-scores. A Moran’s I greater than zero with a Z-score ≥ 1.96 (*p* < 0.05) was considered evidence of statistically significant positive spatial autocorrelation, i.e., a clustered disease distribution. To detect local spatial autocorrelation that may be masked in the global analysis and to explore the presence of spatial heterogeneity, we performed a local spatial autocorrelation analysis using the Anselin Local Moran’s I, a Local Indicator of Spatial Association (LISA) (23). This method identifies spatial units with statistically significant (*p* < 0.05) local clustering or outliers and classifies them into four categories: high-high (HH) and low-low (LL) clusters (hotspots and cold spots, respectively), and high-low (HL) and low-high (LH) spatial outliers (24). Hotspots are generally considered high-priority areas for infectious disease control, and thus they were the primary focus of the spatial analysis of pulmonary tuberculosis in this study.

### 2.4 Forecast evaluation framework

The national monthly dataset (2004–2025) was split into a training period (January 2004–December 2021, 216 months) and a test period (January 2022–December 2025, 48 months). A rolling-window forecasting scheme was adopted to evaluate model performance over successive one-year-ahead forecasts. Starting with the initial training set (2004–2021), each model forecast the next 12 months (one year). The training window was then expanded by adding the actual observations of the just-forecasted year, and the process was repeated, generating one-year-ahead forecasts for 2022, 2023, 2024, and 2025. All analyses were performed using R version 4.4.1 with the packages forecast, prophet, ggplot2, Metrics, zoo, and XGBoost.

Model in-sample fit was compared using the Akaike information criterion (AIC) for SARIMA and Holt-Winters models. Forecast accuracy was assessed using four metrics: mean absolute error (MAE), root mean squared error (RMSE), mean absolute percentage error (MAPE), and symmetric mean absolute percentage error (SMAPE) (25). Let $y_{t}$ denote the observed value and ${\hat{y}}_{t}$ the forecast at time t ( t=1,2,…,n ). The metrics were defined as:


$$\mathrm{MAE}=\frac{1}{n}\sum_{t=1}^{n}|y_{t}-{\hat{y}}_{t}|$$

$$\text{RMSE}=\sqrt{\frac{1}{n}\sum_{t=1}^{n}{\left(y_{t}-{\hat{y}}_{t}\right)}^{2}}$$

$$\text{MAPE}=\frac{100\%}{n}\sum_{t=1}^{n}|\frac{y_{t}-{\hat{y}}_{t}}{y_{t}}|$$

$$\text{SMAPE}=\frac{100\%}{n}\sum_{t=1}^{n}\frac{2|y_{t}-{\hat{y}}_{t}|}{|y_{t}|+|{\hat{y}}_{t}|}$$

For each metric, 95% confidence intervals were obtained by non-parametric bootstrap with 1,000 resamples of the forecast error series. The Akaike information criterion (AIC) was calculated for the Holt-Winters and SARIMA models on the initial training set to compare model parsimony and in-sample fit. The Diebold-Mariano test was used to pairwise compare predictive accuracy, with squared forecast errors as the loss function (26). A significance level of α=0.05 was applied.

Based on the rolling-window performance, the model achieving the lowest forecast errors (MAE, MAPE, and RMSE) was selected as the final model for each outcome. The final model was then refitted to the full dataset (2004–2025) to generate monthly forecasts for 2026–2030 with 95% prediction intervals.

### 2.5 Holt-winters model

The additive Holt-Winters method was used to capture trends and seasonality (27). The model assumes a linear trend and additive seasonal components with a period of 12 months and belongs to the exponential smoothing family, which assigns exponentially decreasing weights to past observations. Three smoothing parameters, *α* (level), *β* (trend), and *γ* (seasonality), each constrained to the interval [0, 1], were estimated by minimizing the sum of squared one-step forecast errors (28).

### 2.6 SARIMA model

The seasonal autoregressive integrated moving average (SARIMA) model extends the ARIMA framework by incorporating seasonal components (29). A SARIMA model is denoted as SARIMA(p,d,q)(P,D,Q)[s], where p, d, and q are the non-seasonal autoregressive, differencing, and moving average orders; P, D, and Q are the corresponding seasonal orders; and s is the number of periods per season (here s = 12 for monthly data) (5). First, auto.arima was applied with stepwise = FALSE and approximation = FALSE for a thorough search over all orders. Second, after applying the indicated differences, the ACF and PACF of the differenced series were visually inspected to confirm the implied seasonal and non-seasonal components. Third, candidate models (including the automatically selected one and alternatives suggested by the ACF/PACF patterns) were compared using AIC and BIC. Fourth, the Ljung-Box test (lag = 24) was used to ensure residuals were white noise. Following this procedure on the last training window (2004–2024), ARIMA(3,0,1)(0,1,1)[12] with drift was identified as the optimal model. It was then refitted to the full dataset (2004–2025) to forecast 2026–2030.

### 2.7 Prophet model

Prophet is a decomposable time series model with trend, seasonality, and holiday components (30). We used the additive seasonality mode with a linear trend, disabling weekly and daily seasonality, as only monthly data were available (31). Instead of using default hyperparameters, we performed a grid search to optimize the two key parameters: changepoint.prior.scale (candidates: 0.01, 0.05, 0.1, 0.5) and seasonality.prior.scale (candidates: 0.1, 1, 5, 10). Using the initial training set (2004–2021) with the last 12 months as a validation set, we selected the combination that minimized the mean absolute percentage error (MAPE) on the validation set. The optimal hyperparameters were then fixed for all rolling windows and for the final model refitted to the full dataset (2004–2025). Prediction intervals were set to 95% using interval.width = 0.95.

### 2.8 XGBoost model

XGBoost (eXtreme Gradient Boosting) is a scalable tree-based ensemble algorithm (32). To adapt it for time series forecasting, we used a supervised learning formulation based on lagged values: for each prediction step, the 12 most recent monthly incidence (or mortality) rates (lags 1 to 12) were used as features to predict the next month’s rate. The model was trained with squared error loss (reg:squarederror). Hyperparameters were set as eta = 0.1, max_depth = 5, subsample = 0.8, colsample_bytree = 0.8, and min_child_weight = 3. Early stopping with 10 rounds of patience was applied on a validation set consisting of the last 12 months of the training period (33). The same rolling-window scheme described in the Forecast evaluation framework was used to produce one-year-ahead forecasts for 2022–2025. Predictions were made recursively: starting from the last 12 observed values, each predicted month was fed back as input for the next prediction. To obtain probabilistic forecasts, we employed quantile regression using the reg:quantileerror objective. Separate models were trained for the 2.5th, 50th (median), and 97.5th percentiles, providing 95% prediction intervals. The final models for both incidence and mortality were refitted to the full dataset (2004–2025) using the same hyperparameters, and forecasts for 2026–2030 were generated with 95% prediction intervals.

## 3 Results

### 3.1 Overview of PTB in China between 2004 and 2023

**Figure 1:**
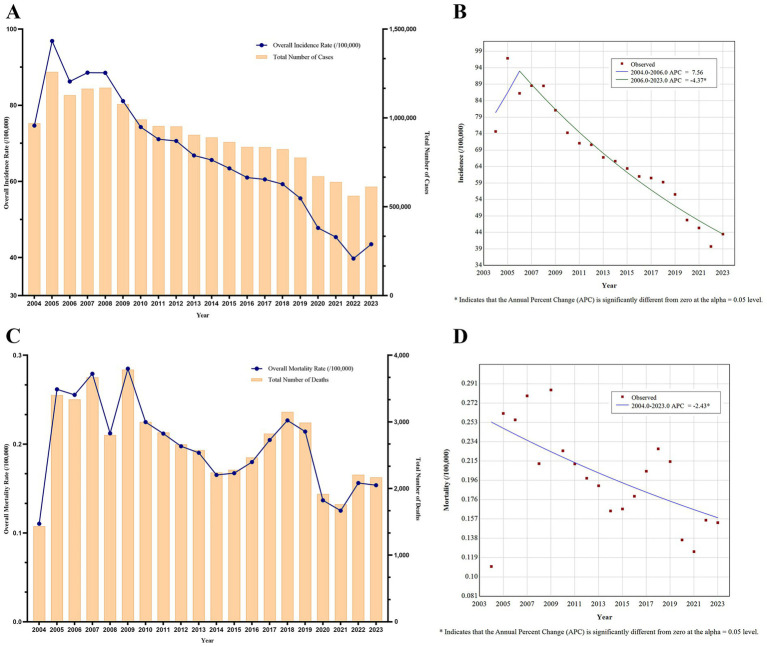
Temporal trends of PTB incidence and mortality in China, 2004–2023. **(A,B)** Trends in incidence rates. **(C,D)** Trends in mortality rates. Joinpoint regression was used to identify significant changes in trends. The annual percent change (APC) and its 95% confidence interval (CI) were calculated.

Between 2004 and 2023, the average annual incidence rate of pulmonary tuberculosis in China was approximately 66.23 per 100,000 population, while the average annual mortality rate was 0.20 per 100,000. Figure 1 illustrates the annual fluctuations in new cases and deaths over the two decades. The number of new cases closely mirrored the incidence rate. Both peaked in 2005 (1,259,308 cases; 96.88 per 100,000), then declined substantially to their lowest levels in 2022 (560,847 cases; 39.73 per 100,000). By 2023, the incidence rate had decreased to 41.0% of the 2005 peak and the number of cases to 44.5% (Figure 1A). Similarly, the number of deaths tracked the mortality rate. Both peaked in 2009 (3,783 deaths; 0.28 per 100,000). After a fluctuating decline, they reached a nadir in 2021 (1,763 deaths; 0.13 per 100,000) (Figure 1C). Joinpoint regression identified 2006 as a significant turning point for incidence. From 2004 to 2005, the incidence showed a non-significant increase (APC = 7.56%, *p* > 0.05), followed by a sustained downward trend from 2006 to 2023 (APC = −4.37, 95% CI: −7.60 to −3.86%, *p* < 0.05) (Figure 1B). For mortality, the overall trend from 2004 to 2023 was decreasing (APC = −2.43, 95% CI: −4.12 to −0.71%, p < 0.05), with no statistically significant joinpoints detected (Figure 1D).

### 3.2 Spatio-temporal distribution of PTB incidence and mortality in China, 2004–2023

**Figure 2:**
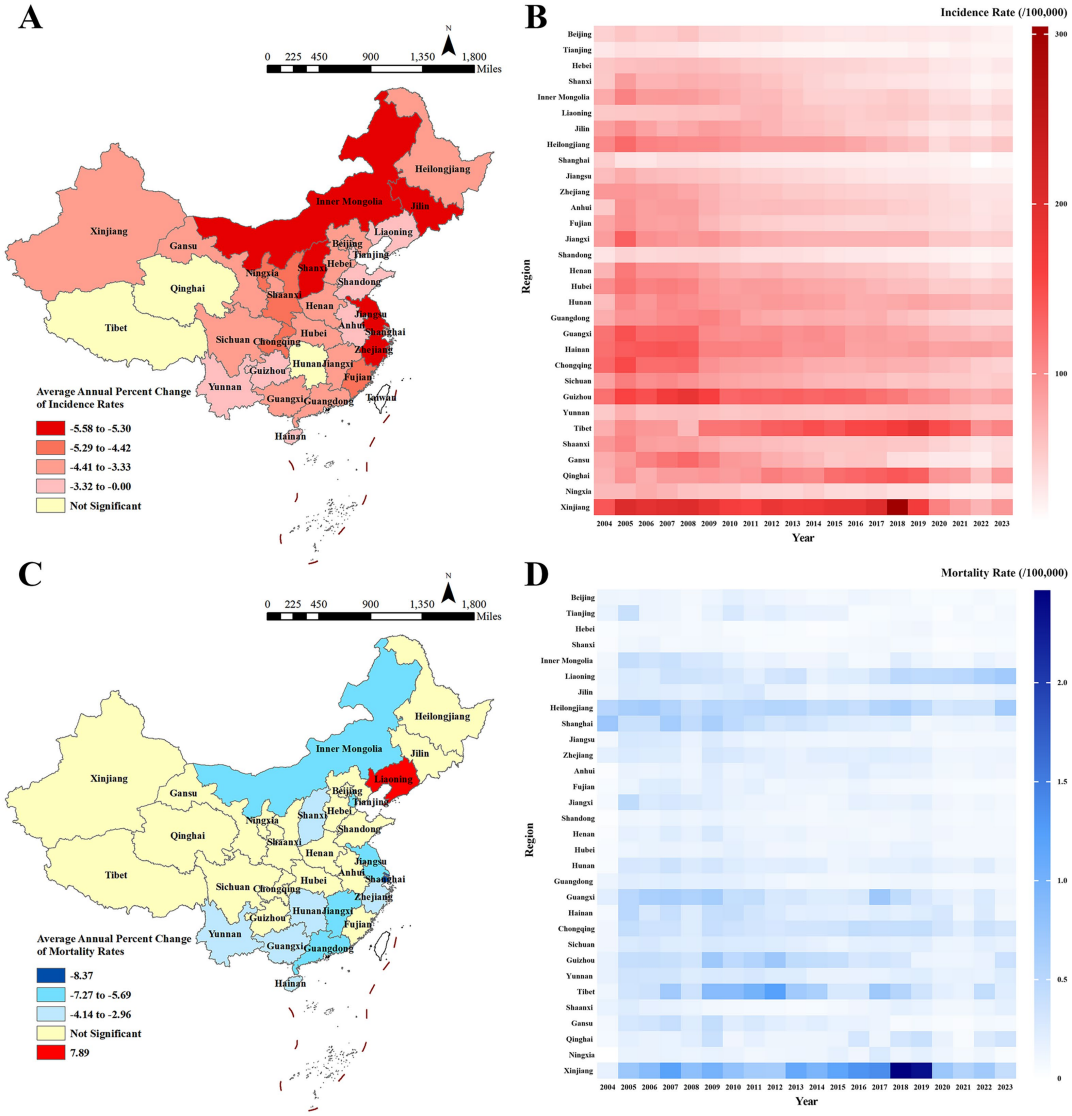
Spatial distribution of average annual percentage change (AAPC) and temporal heatmaps of PTB incidence and mortality in China, 2004–2023. **(A)** Map of AAPC in incidence. Except for three provinces (Tibet, Hunan, and Qinghai), the remaining 28 provinces showed a statistically significant decreasing trend (*p* < 0.05). The fastest declines were observed in three northern provinces (Inner Mongolia, Jilin, and Shanxi) and two eastern coastal provinces (Jiangsu and Zhejiang). **(B)** Heatmap of annual incidence time series (31 provinces). Each row represents a province; color intensity reflects the incidence rate over time. **(C)** Map of AAPC in mortality. Only 12 provinces exhibited a significant decreasing trend: Yunnan, Guangxi, Guangdong, Hunan, Jiangxi, Hainan, Jiangsu, Shanghai, Zhejiang, Tianjin, Shanxi, and Inner Mongolia. The fastest decline occurred in Shanghai. Notably, only Liaoning Province showed a significant increasing trend. **(D)** Heatmap of annual mortality time series (31 provinces). Each row represents a province; color intensity reflects the mortality rate over time. AAPC and its significance were estimated using joinpoint regression analysis (*p* < 0.05).

Figure 2 illustrates the distribution of the PTB burden across provinces in mainland China, revealing significant regional variations in the annual average percentage change (AAPC) in PTB incidence between 2004 and 2023. With the exception of three provinces in central and western China (Tibet, Hunan, and Qinghai), where the AAPC in incidence was not statistically significant, the remaining 28 provinces in China all exhibited a significant downward trend in incidence. Among these, three provinces in northern China (Inner Mongolia: AAPC = −5.53; Jilin AAPC = −5.58; Shanxi AAPC = −5.57) and the two eastern coastal provinces (Jiangsu AAPC = −5.53; Zhejiang AAPC = −5.30) exhibited the fastest rates of decline in incidence (Figure 2A).

In most regions of mainland China (19 provinces), the AAPC for PTB mortality was not significant. Only 12 provinces—in southern China (Yunnan, Guangxi, Guangdong, Hunan, Jiangxi, and Hainan), the eastern coastal region (Jiangsu, Shanghai, and Zhejiang), and the north (Tianjin, Shanxi, and Inner Mongolia)—exhibited a significant downward trend in mortality rates. Among these, the province with the fastest rate of decline was the eastern coastal city of Shanghai (AAPC = −8.37, *p* < 0.001). Notably, only Liaoning Province showed a significant upward trend in mortality (AAPC = 7.89, *p* < 0.001) (Figure 2C) (for detailed AAPCs of incidence and mortality by province, see Supplementary Figures S1, S2).

Between 2004 and 2023, among the 31 provinces and municipalities, the four regions in western China—Xinjiang, Qinghai, Tibet, and Guizhou—exhibited higher rates of pulmonary tuberculosis, with Xinjiang being the most prominent. Incidence and mortality rates in Xinjiang remained persistently high, with incidence peaking in 2018 and mortality peaking in 2018–2019. Apart from Xinjiang, Tibet also exhibited high mortality rates during the period 2009–2014 (Figures 2B,D).

### 3.3 Spatial autocorrelation of PTB incidence in China, 2004–2023

Global spatial autocorrelation analysis revealed that annual provincial PTB incidence rates were positively and significantly spatially autocorrelated across all years from 2004 to 2023, with Moran’s I values ranging from 0.206 to 0.580 (all *p* < 0.05) (Table 1). This indicates a consistently clustered spatial pattern of PTB incidence over the study period.

**Table 1:** Global Moran’s I index for spatial association of PTB incidence, 2004–2023.

Year	Moran’s I	Z-score	*p*	Pattern
2004	0.206	2.022	0.043^*^	Clustered
2005	0.254	2.470	0.014^*^	Clustered
2006	0.228	2.283	0.022^*^	Clustered
2007	0.284	2.768	0.006^*^	Clustered
2008	0.234	2.342	0.019^*^	Clustered
2009	0.339	3.284	0.001^*^	Clustered
2010	0.385	3.642	0.000^*^	Clustered
2011	0.447	4.145	0.000^*^	Clustered
2012	0.467	4.412	0.000^*^	Clustered
2013	0.480	4.500	0.000^*^	Clustered
2014	0.457	4.325	0.000^*^	Clustered
2015	0.470	4.437	0.000^*^	Clustered
2016	0.503	4.771	0.000^*^	Clustered
2017	0.517	4.999	0.000^*^	Clustered
2018	0.397	4.619	0.000^*^	Clustered
2019	0.531	5.073	0.000^*^	Clustered
2020	0.538	5.089	0.000^*^	Clustered
2021	0.523	4.964	0.000^*^	Clustered
2022	0.520	4.716	0.000^*^	Clustered
2023	0.580	5.228	0.000^*^	Clustered

* indicates the corresponding significance level (*p*-value) is < 0.05. This table presents the annual global Moran’s I values, Z-scores, *p*-values, and spatial patterns for pulmonary tuberculosis (PTB) incidence across Chinese provinces from 2004 to 2023. All Moran’s I values were positive and statistically significant (*p* < 0.05), indicating consistent positive spatial autocorrelation: i.e., provinces with similar incidence levels (high with high, low with low) tended to cluster geographically. The analysis was performed using ArcGIS 10.8, with statistical significance assessed via Z-scores (critical value ±1.96 for *p* < 0.05).

**Figure 3:**
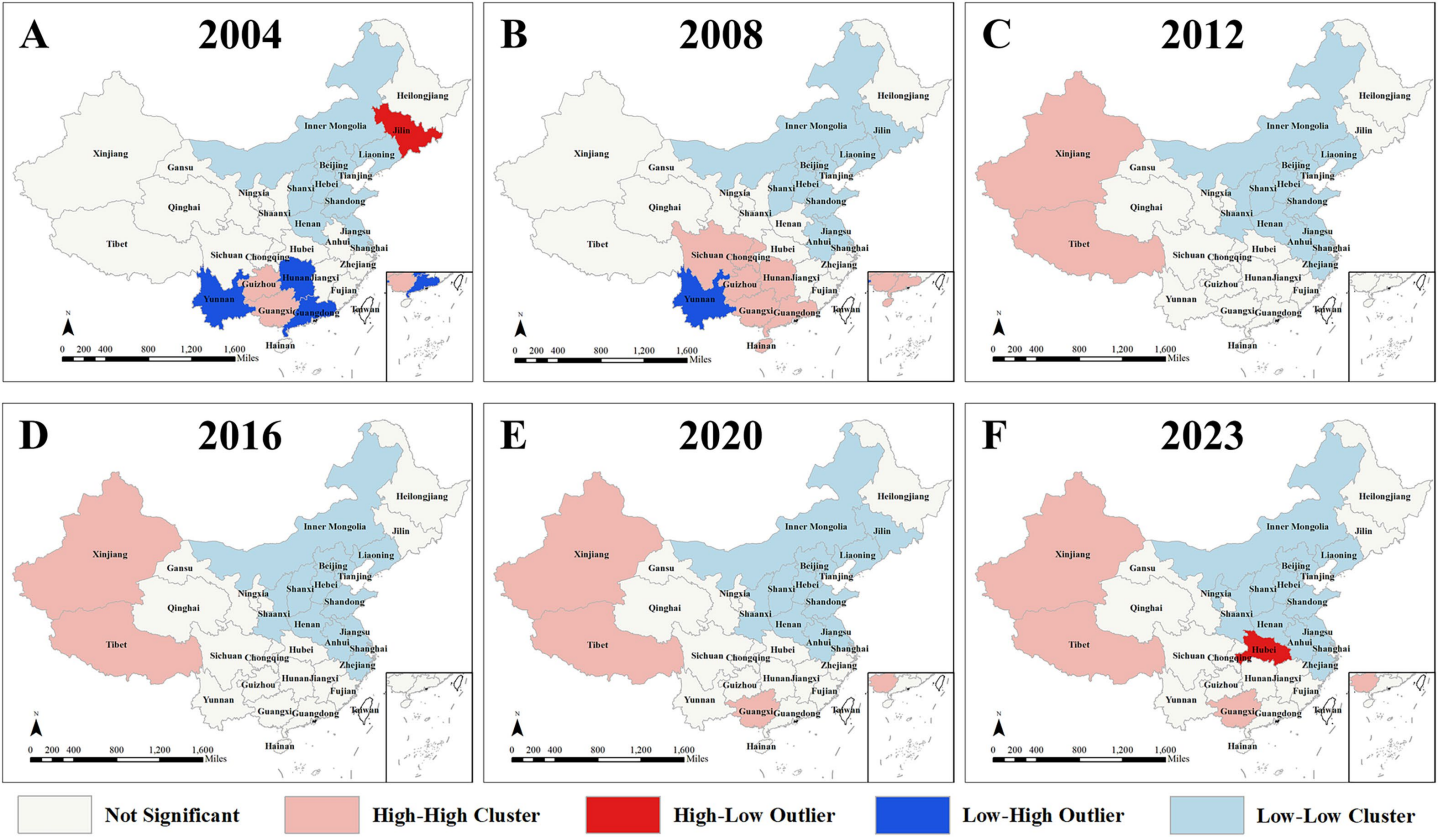
Spatial distribution of local clustering patterns of PTB incidence across 31 provinces in mainland China, 2004–2023. The figure presents local spatial autocorrelation results for six selected years [2004 **(A)**, 2008 **(B)**, 2012 **(C)**, 2016 **(D)**, 2020 **(E)**, and 2023 **(F)**], which were chosen to approximately divide the entire 20-year study period into six equal temporal segments to capture the spatiotemporal evolution of PTB incidence. Clustering types were identified using the Anselin Local Moran’s I statistic (Local Indicators of Spatial Association, LISA) and classified into four categories: High-High (HH): provinces with high incidence surrounded by high-incidence neighbors (hotspots); Low-Low (LL): provinces with low incidence surrounded by low-incidence neighbors (cold spots); High-Low (HL): provinces with high incidence surrounded by low-incidence neighbors (spatial outliers); Low-High (LH): provinces with low incidence surrounded by high-incidence neighbors (spatial outliers). Only spatial units with statistically significant clustering (*p* < 0.05) are shown. Non-significant areas are displayed as grey.

Local spatial autocorrelation analysis using Anselin local Moran’s I further identified significant (*p* < 0.05) spatial clusters and outliers. The spatiotemporal evolution of these categories is summarized as follows (Figure 3). High-High (HH) clusters (hotspots) were primarily located in southern China (Guizhou, Guangxi, Hunan, Chongqing, Guangdong, Sichuan, and Hainan) between 2004 and 2008. After 2008, except for Guangxi, these HH clusters gradually dissipated in southern China and reappeared in western China (Tibet and Xinjiang). Low-Low (LL) clusters (cold spots) initially centered on North China (Beijing, Tianjin, Hebei, Shanxi, and Inner Mongolia), Northeast China (Liaoning), Central China (Henan), and East China (Shandong and Jiangsu). Over time, they expanded northwestward (Shaanxi and Ningxia) and southeastward (Anhui, Shanghai, and Zhejiang), showing a divergent spread toward the central-western regions and the Yangtze River Delta. High-Low (HL) outliers shifted from Jilin in Northeast China to Hubei Province in Central China during the study period. Low-High (LH) outliers were observed in southern China (Hunan, Yunnan, and Guangdong) from 2004 onwards. These areas gradually transitioned into HH clusters and subsequently became non-significant (i.e., no longer showing statistically significant local spatial autocorrelation).

### 3.4 Incidence and mortality forecasts to 2030

Table 2 presents the rolling-window forecast errors for incidence and mortality. The rankings of error metrics (MAE, MAPE, RMSE) and the Diebold-Mariano tests (Table 3) identified the best-performing model for each outcome. The candidate SARIMA model comparison (Supplementary Table S1) confirmed that the selected ARIMA(3,0,1)(0,1,1)[12] with drift had the lowest AIC and acceptable residual diagnostics. The autocorrelation function (ACF) and partial autocorrelation function (PACF) of the differenced series used for SARIMA model identification are shown in Supplementary Figure S3 (incidence) and Supplementary Figure S4 (mortality), supporting the selected seasonal and non-seasonal orders. The rolling forecast comparisons for incidence and mortality are provided in the Supplementary Figures S5, S6.

**Table 2:** Model performance: out-of-sample forecast errors and in-sample fit.

Model	MAE	MAPE	SMAPE	RMSE	AIC
Incidence					
Holt-Winters	0.93	22.93	21.74	1.04	870.55
SARIMA	0.65	16.13	15.47	0.79	270.23
Prophet	0.55	13.38	13.35	0.67	NA
XGBoost	0.49	12.72	11.49	0.65	NA
Mortality					
Holt-Winters	0.0065	30.42	34.58	0.0084	−1265.92
SARIMA	0.0064	30.28	35.20	0.0084	−1822.06
Prophet	0.0096	45.98	53.87	0.0109	NA
XGBoost	0.0050	23.22	26.46	0.0066	NA

Rolling-window forecast errors for incidence and mortality based on expanding training windows (starting from 2004–2021 up to 2004–2024) to produce one-year-ahead forecasts for each year from 2022 to 2025. Values shown are point estimates. Metrics: mean absolute error (MAE), mean absolute percentage error (MAPE, %), symmetric mean absolute percentage error (SMAPE, %), and root mean square error (RMSE). AIC (Akaike information criterion) is an in-sample fit measure calculated on the initial training set (2004–2021) for the Holt-Winters (additive error, additive trend, additive seasonality; implemented via the ets function in R) and SARIMA models and is not a forecast accuracy metric. AIC is not applicable (NA) for Prophet and XGBoost. For incidence, XGBoost achieved the lowest forecast errors (MAE = 0.49, MAPE = 12.72%) and was selected as the final forecasting model. For mortality, XGBoost also yielded the lowest errors (MAE = 0.0050, MAPE = 23.22%) and was selected as the final model. All analyses were performed using R 4.4.1 with packages forecast (Holt-Winters, SARIMA), prophet, and xgboost. Bootstrap 95% confidence intervals (1,000 resamples) are available but not shown.

**Table 3:** Diebold-Mariano test comparisons of predictive accuracy.

Comparison	Incidence		Mortality	
	DM Statistic	*p*	DM Statistic	*p*
Holt-winters vs SARIMA	2.84	0.01^*^	−0.76	0.45
Holt-winters vs Prophet	2.62	0.01^*^	−2.89	0.01^*^
Holt-winters vs XGBoost	2.89	0.01^*^	3.61	0.00^*^
SARIMA vs Prophet	1.12	0.27	−3.04	0.00^*^
SARIMA vs XGBoost	0.35	0.73	3.77	0.00^*^
Prophet vs XGBoost	−0.57	0.57	3.92	0.00^*^

* indicates the corresponding significance level (*p*-value) is < 0.05. Pairwise comparisons of forecast accuracy using the Diebold-Mariano test with squared forecast errors as the loss function (one-step-ahead horizon). Values are the DM test statistic and the corresponding two-sided *p*-value. For incidence, XGBoost achieved the lowest point forecast errors (MAE = 0.49, MAPE = 12.72%), although the DM test did not detect statistically significant differences between XGBoost and SARIMA (*p* = 0.73) or between XGBoost and Prophet (*p* = 0.57). For mortality, XGBoost significantly outperformed all other models (all *p* < 0.001), consistent with its lowest forecast errors (MAE = 0.0050, MAPE = 23.22%). The test was implemented using the forecast package in R version 4.4.1.

**Figure 4:**
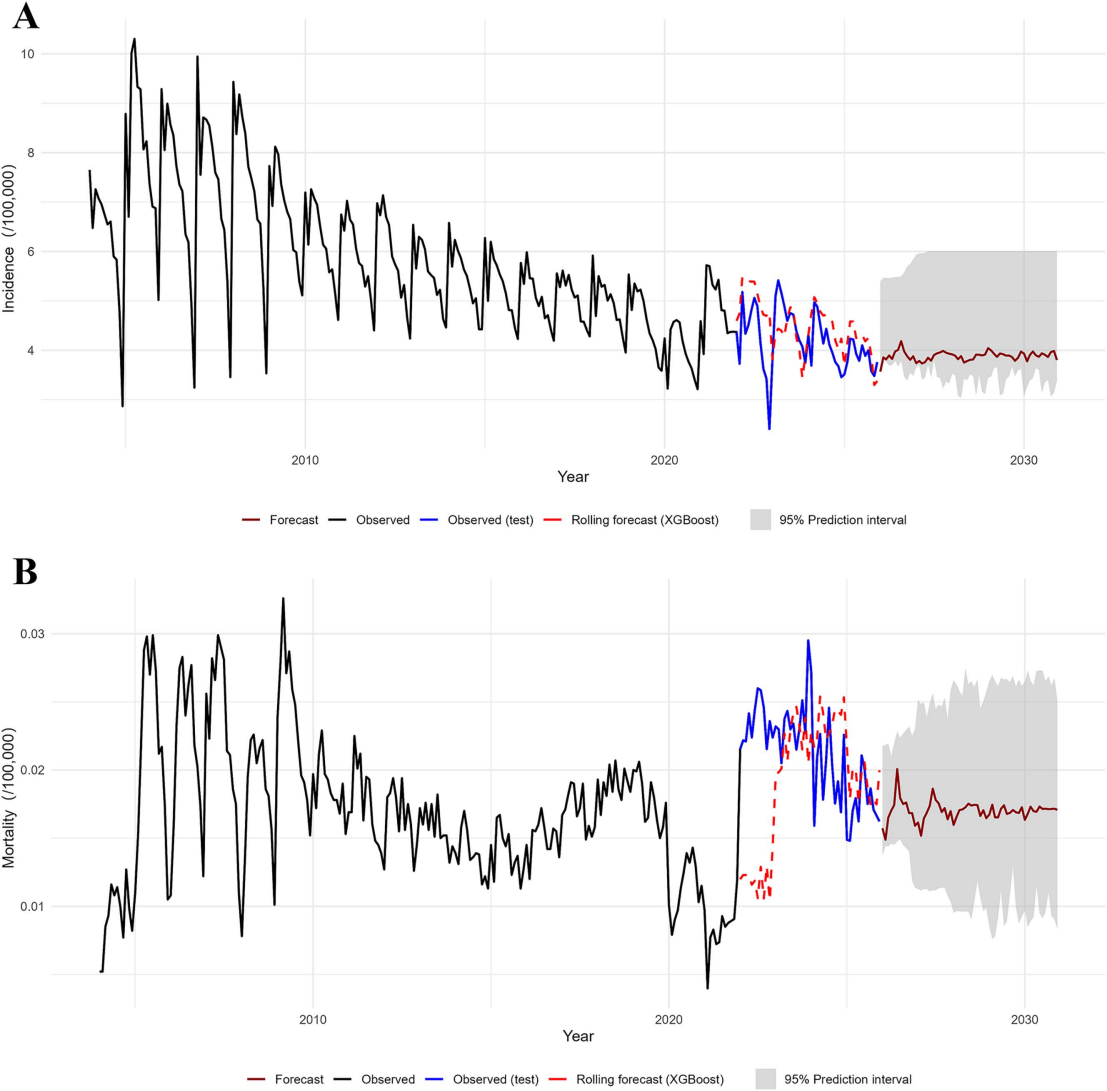
Forecasted trends of PTB incidence and mortality in China based on rolling-window validation and final projections to 2030. **(A)** Incidence: forecasts generated by the XGBoost model using quantile regression (50th percentile as a point forecast and 2.5th and 97.5th percentiles for 95% prediction intervals). The model was evaluated using an expanding rolling-window scheme (training windows from 2004–2021 to 2004–2024) to produce one-year-ahead forecasts for 2022–2025 (rolling forecast), and then refitted to the full dataset (2004–2025) to project monthly incidence from 2026 to 2030. Hyperparameters: eta = 0.1, max_depth = 5, subsample = 0.8, colsample_bytree = 0.8, min_child_weight = 3. Observed: historical observed values (2004–2021). Observed (test): actual observed values during the test period (2022–2025). Rolling forecast: rolling forecast values (one-year-ahead predictions for 2022–2025). Forecast: final forecast values (2026–2030). Shaded area: 95% prediction interval (from XGBoost quantile regression). **(B)** Mortality: forecasts generated by the XGBoost model using quantile regression (same settings as for incidence). The same rolling-window evaluation was applied, and the final model was refitted to the full dataset to produce projections for 2026–2030 with 95% prediction intervals. Observed: historical observed values (2004–2021). Observed (test): actual observed values during the test period (2022–2025). Rolling forecast: rolling forecast values (one-year-ahead predictions for 2022–2025). Forecast: final forecast values (2026–2030). Shaded area: 95% prediction interval (from XGBoost quantile regression).

For incidence, XGBoost achieved the lowest forecast errors (MAE = 0.49, MAPE = 12.72%). Although the Diebold-Mariano test showed no statistically significant differences between XGBoost and SARIMA (*p* = 0.73) or between XGBoost and Prophet (*p* = 0.57, Table 3), XGBoost consistently produced the smallest point forecast errors and was therefore selected as the final forecasting model. Using the full dataset (2004–2025) and quantile regression, the XGBoost model projects that from 2026 to 2030, the monthly incidence will plateau at approximately 3.5–4.2 per 100,000 with mild seasonal fluctuations, rather than continuing the sharp downward trend observed in previous decades. The 95% prediction intervals widen moderately over time (upper bound ~6.0 after 2028), reflecting some uncertainty but no indication of a cyclical rebound (Figure 4A). Detailed monthly forecasts are provided in Supplementary Table S2.

For mortality, XGBoost also outperformed all other models, with the lowest MAE (0.0050), MAPE (23.22%), and RMSE (0.0066). The Diebold-Mariano test confirmed that XGBoost significantly outperformed Holt-Winters, SARIMA, and Prophet (all *p* < 0.001, Table 3). The final XGBoost model predicts that mortality rates will remain stable at a very low level (0.015–0.021 per 100,000) throughout 2026–2030, with no sustained upward or downward trend. The 95% prediction intervals are relatively wide (0.008–0.027), indicating greater uncertainty in mortality projections to 2030, but the risk of a resurgence appears minimal (Figure 4B). Monthly forecasts are available in Supplementary Table S3.

## 4 Discussion

The present study provides a comprehensive analysis of the temporal trends, spatial dynamics, and future trajectories of pulmonary tuberculosis in China based on a consistent national surveillance dataset spanning 2004–2025. Unlike most previous studies that focused either on short-term trends or on single-region analyses, the present work integrates long-term national-level and provincial-level data, identified a westward shift of hotspots after 2008, and uncovered a paradoxical rise in mortality in Liaoning Province despite declining incidence and persistently low spatial risk. Moreover, the forecasting comparison demonstrates that the machine learning model XGBoost achieved the lowest forecast errors for both incidence and mortality, while traditional models (SARIMA, Prophet) also performed reasonably well, providing a benchmark for routine surveillance. Comparison with recent national-level TB forecasting studies (34, 35) shows that our work extends the literature by employing a rolling-window validation framework, simultaneously forecasting both incidence and mortality, introducing XGBoost with quantile regression to obtain probabilistic 95% prediction intervals, and projecting an incidence plateau (3.5–4.2/100,000) rather than a continued decline.

The results show a sustained and substantial decline in both PTB incidence and mortality over the past two decades. This decline was particularly notable after 2005, when PTB notifications peaked, and was driven by the nationwide implementation of the Directly Observed Treatment, Short-course (DOTS) strategy, which has been a cornerstone of global TB control efforts (7, 10) The DOTS strategy, combined with increased government funding for infectious disease surveillance and improved laboratory diagnostics, significantly enhanced case detection and treatment success rates (8). This progress was further bolstered by the integration of TB services into primary healthcare systems, ensuring broader access to diagnosis and treatment, particularly in rural and high-burden regions (36). Technological and policy innovations also played a pivotal role. The establishment of the Tuberculosis Information Management System (TBIMS) in 2005 enabled real-time monitoring of TB cases, improving the accuracy and timeliness of epidemiological data and facilitating the identification of high-risk groups and regions for more efficient resource allocation (9, 15).

Despite the overall progress, several distinct geographical anomalies warrant attention. At the national level, the only province showing a significant upward trend in PTB mortality was Liaoning. This increase occurred even though Liaoning’s incidence declined and local spatial autocorrelation analysis consistently identified the province as a cold spot (low-low cluster) throughout the study period. This paradox — rising mortality despite falling incidence and low spatial risk — suggests that factors other than transmission intensity may be driving fatal outcomes in this northeastern industrial region. Liaoning’s aging population likely exacerbates TB mortality, as older adults face higher risks of TB reactivation due to immunosenescence and comorbid conditions such as chronic obstructive pulmonary disease (COPD), diabetes, and silicosis — an occupational hazard in industrial areas (6, 37). A meta-analysis confirmed that silicosis patients have a four-fold increased risk of tuberculosis (RR = 4.01, 95% CI: 2.88–5.58) (38). Post-TB complications, particularly COPD, have been shown to significantly contribute to mortality in TB patients, especially among older males (11). Delayed diagnosis, which is more common in resource-limited settings, has also been associated with worse outcomes (9). Despite Liaoning’s “cold spot” status, localized healthcare inequities — such as uneven access to diagnostics or suboptimal management of drug-resistant TB (DR-TB) — may contribute to the observed mortality increase. The industrial legacy of the province may further intersect with occupational lung diseases (e.g., silicosis) and air pollution to exacerbate TB severity (12, 39). Liaoning’s rising TB mortality amid declining incidence signals a need to shift focus from transmission control to comprehensive, patient-centered care. By addressing aging-related vulnerabilities, comorbidities, and systemic inequities, policymakers can bridge the gap between low spatial risk and high mortality — a critical step toward achieving China’s 2030 TB elimination targets (40). Future research should quantify the contributions of silicosis and air pollution to TB fatalities in industrial settings, while pilot interventions test the scalability of integrated care models.

In stark contrast, Shanghai exhibited the fastest decline in mortality among all provinces, reflecting the benefits of a well-resourced healthcare system and effective case management (13).

Meanwhile, although Xinjiang showed a statistically significant downward trend in incidence, its incidence and mortality rates remained persistently higher than those of most other provinces throughout 2004–2023, with incidence peaking as late as 2018. Previous studies have confirmed Xinjiang as a persistent high-burden area, with reported incidence rates among the highest in China (4, 41). Spatial autocorrelation analysis further revealed a marked shift of high-high (HH) clusters (hotspots) from southern China in the early 2000s to western China—specifically Xinjiang and Tibet—after 2008. Tibet’s high altitude (≈4,000 m) is an established risk factor for TB (42). A nationwide spatial analysis (2013–2016) identified TB hotspots in Xinjiang and Tibet, associated with lower rural income, fewer health personnel, and higher illiteracy rates (43). A longitudinal study in Xinjiang (2005–2019) reported an average annual incidence of 172/100,000, with low GDP per capita, high PM₂.₅, and low health workforce as the top three risk factors (44). Studies have similarly demonstrated that the average annual hotspot regions for PTB incidence in China are Tibet and Xinjiang, consistent with a westward shift of the disease burden (14, 16). This indicates that western China remains the primary burden region. This westward movement mirrors the uneven distribution of socioeconomic development, healthcare access, and poverty rates (45, 46). Western regions, characterized by lower population density, limited medical resources, and higher proportions of ethnic minority populations with different healthcare-seeking behaviors (47), have become the new epicenters of PTB transmission. Similar spatial patterns have been documented in other large countries such as Brazil and Russia, where internal migration and regional economic disparities drive TB epidemiology (48, 49). In Russia, TB incidence varies considerably across geographic regions, with the highest rates occurring in the eastern portion of the country, which faces similar challenges of territorial remoteness and resource limitation (48, 50). In Brazil, spatiotemporal clustering analyses have identified TB hotspots among international migrants in the northern states, demonstrating how internal migration and socioeconomic disparities shape TB transmission dynamics (49). These findings underscore the need for geographically tailored interventions that address not only transmission but also comorbidities and healthcare system performance in specific provinces (51).

The XGBoost model projects a plateau in incidence (approximately 3.5–4.2 per 100,000) rather than a continued decline, suggesting that China’s downward trend over the past two decades may have stabilized. Mortality is predicted to remain at a very low level, though wide prediction intervals underscore the inherent uncertainty in forecasting rare events. Although the Diebold-Mariano test revealed no significant differences in incidence forecasting among XGBoost, SARIMA, and Prophet, XGBoost was selected for its consistently smallest point errors; for mortality, XGBoost was clearly superior. Overall, the historical time series is highly stable, and even simpler models yield reasonable predictions, which is reassuring for routine surveillance. This stability itself is a positive signal, suggesting that China’s TB epidemic has entered a low-level, controlled phase (1). However, the wide prediction intervals for mortality highlight considerable uncertainty in 5-year projections, primarily due to the low absolute mortality counts (rather than rates) and potential underreporting (52). To meet the WHO End TB Strategy’s 2030 milestones—an 80% reduction in incidence and a 90% reduction in mortality from 2015 levels—China must accelerate its current pace of decline, particularly in high-burden western provinces and the emerging anomaly in Liaoning (53). Future efforts should prioritize the management of multidrug-resistant TB and leverage emerging technologies, such as long-read sequencing, to better understand transmission dynamics in high-burden and emerging hotspot regions (54, 55).

The practical significance of this study is threefold. First, it provides evidence-based support for geographically targeted interventions: western hotspots (Xinjiang, Tibet) and the emerging Liaoning anomaly should receive prioritized resources, including active case finding, mobile screening units, and culturally adapted health education. Second, although XGBoost achieved the lowest point errors, the comparable performance of SARIMA and Prophet for incidence (DM test *p* > 0.05) suggests that parsimonious models remain useful for routine surveillance, especially when computational resources are limited. Third, the long-term downward trend documented here offers a benchmark for evaluating the impact of future policy changes or external shocks, such as the future recovery of TB services.

## 5 Conclusion

In conclusion, pulmonary tuberculosis incidence and mortality in China have declined substantially over the past two decades, but significant geographic disparities persist, with western provinces remaining hotspots and Liaoning showing a concerning mortality increase. XGBoost, the best-performing model, projects that incidence will plateau at approximately 3.5–4.2 per 100,000 through 2030 rather than continue its historical decline, while mortality is expected to remain stable at a very low level (≈0.017 per 100,000). These findings underscore the need for geographically targeted interventions, sustained surveillance, and further investigation into the causes of regional anomalies. Although machine learning models such as XGBoost achieved the highest accuracy, traditional models (SARIMA and Prophet) also produced reasonable predictions, suggesting that a balanced approach prioritizing high-quality data collection while leveraging advanced analytics when feasible is appropriate. Accelerating progress toward TB elimination in China will require addressing the root causes of regional heterogeneity and preparing for potential disruptions to the healthcare system.

## 6 Limitations

Several limitations must be acknowledged. First, the data were aggregated at the provincial or national level, which may mask within-province heterogeneity, particularly between urban and rural areas. Second, provincial data were only available up to 2023, so the most recent spatial dynamics (2024–2025) could not be assessed. Third, the forecasting models did not incorporate potential predictors of PTB transmission, such as population density, air quality, migration flows, HIV co-infection rates, or drug-resistance patterns. Inclusion of such covariates could improve forecast accuracy and help explain regional differences. Fourth, the XGBoost quantile regression produced prediction intervals that became constant for incidence after 2028 (upper bound ∼6.0), reflecting a saturation effect inherent to tree-based recursive forecasting; this may underestimate uncertainty in the distant future. Moreover, despite our grid search, the selected hyperparameters may not be globally optimal. Finally, 5-year forecasts (to 2030) are subject to increasing uncertainty, and the projections should be interpreted as possible trajectories rather than precise predictions.

## Data Availability

The original contributions presented in the study are included in the article/Supplementary material, further inquiries can be directed to the corresponding author.

## Footnotes

1
https://www.phsciencedata.cn

2
http://www.nhc.gov.cn

3
https://www.ndcpa.gov.cn

4
https://www.tianditu.gov.cn
