# Supplementary material for: Trends and spatial distribution of pulmonary tuberculosis in China: a surveillance study
Source: Front Public Health. 2026 Jul 1;14:1866155. doi: 10.3389/fpubh.2026.1866155 (PMC13369445; doi:10.3389/fpubh.2026.1866155)
Supplement: Supplementary file 1 [file Data_Sheet_1.DOCX]

**Supplementary Figure S1.** Forest plot of average annual percent change (AAPC) in pulmonary tuberculosis incidence across 31 provinces in China, 2004-2023.
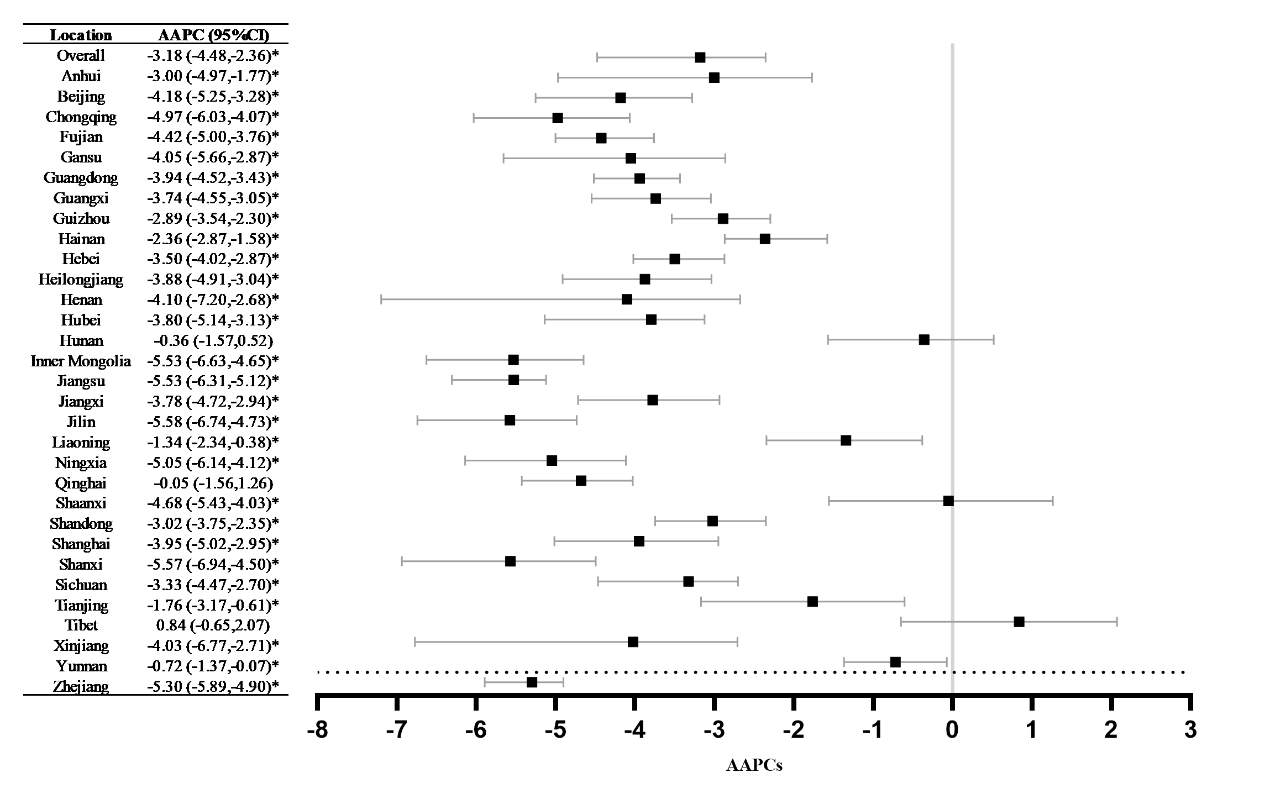


Note: Forest plot showing the provincial average annual percent change (AAPC) in pulmonary tuberculosis incidence with 95% confidence intervals. Estimates were derived from Joinpoint regression (log-linear model, weighted BIC, empirical quantile method). Squares represent point estimates; horizontal lines indicate 95% CIs. The vertical dashed line indicates no change (AAPC = 0).

**Supplementary Figure S2.** Forest plot of average annual percent change (AAPC) in pulmonary tuberculosis mortality across 31 provinces in China, 2004-2023.
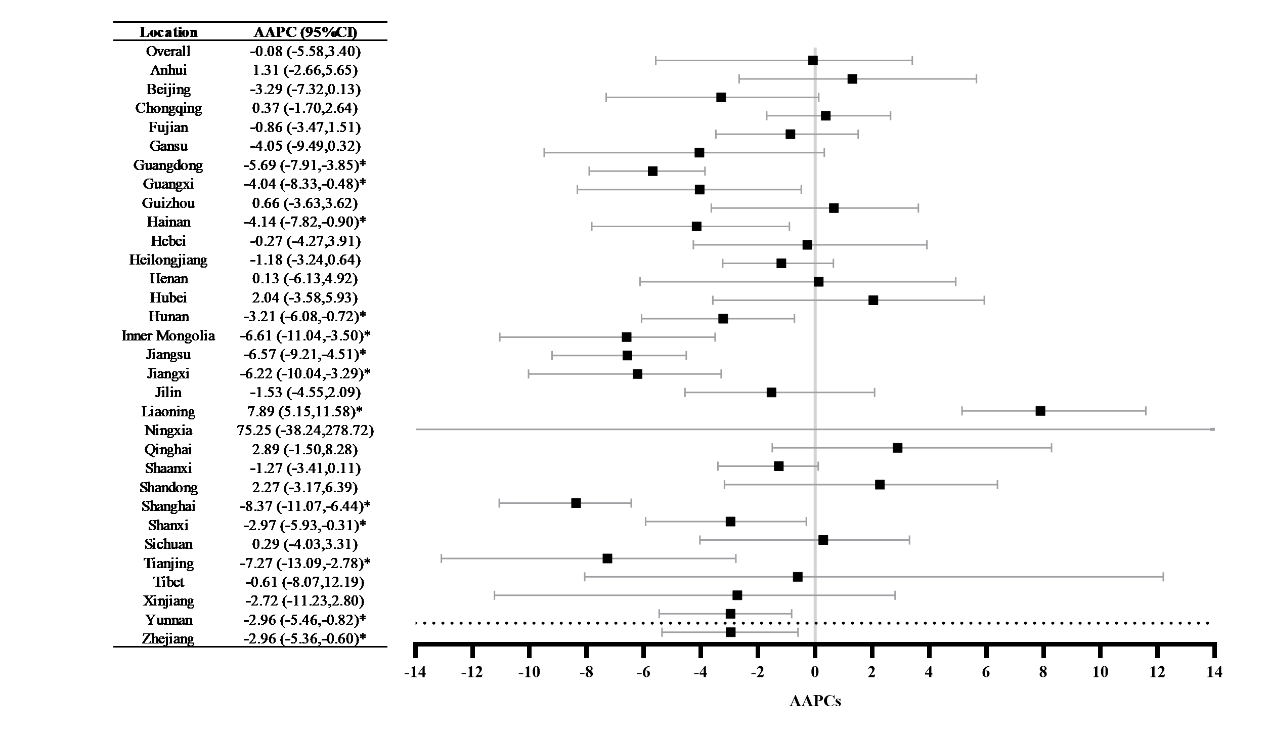


Note: Forest plot showing the provincial average annual percent change (AAPC) in pulmonary tuberculosis mortality with 95% confidence intervals. Estimates were derived from Joinpoint regression (log-linear model, weighted BIC, empirical quantile method). Squares represent point estimates; horizontal lines indicate 95% CIs. The vertical dashed line indicates no change (AAPC = 0).

**Supplementary Figure S3**. ACF and PACF of the seasonally differenced incidence series, 2004-2024


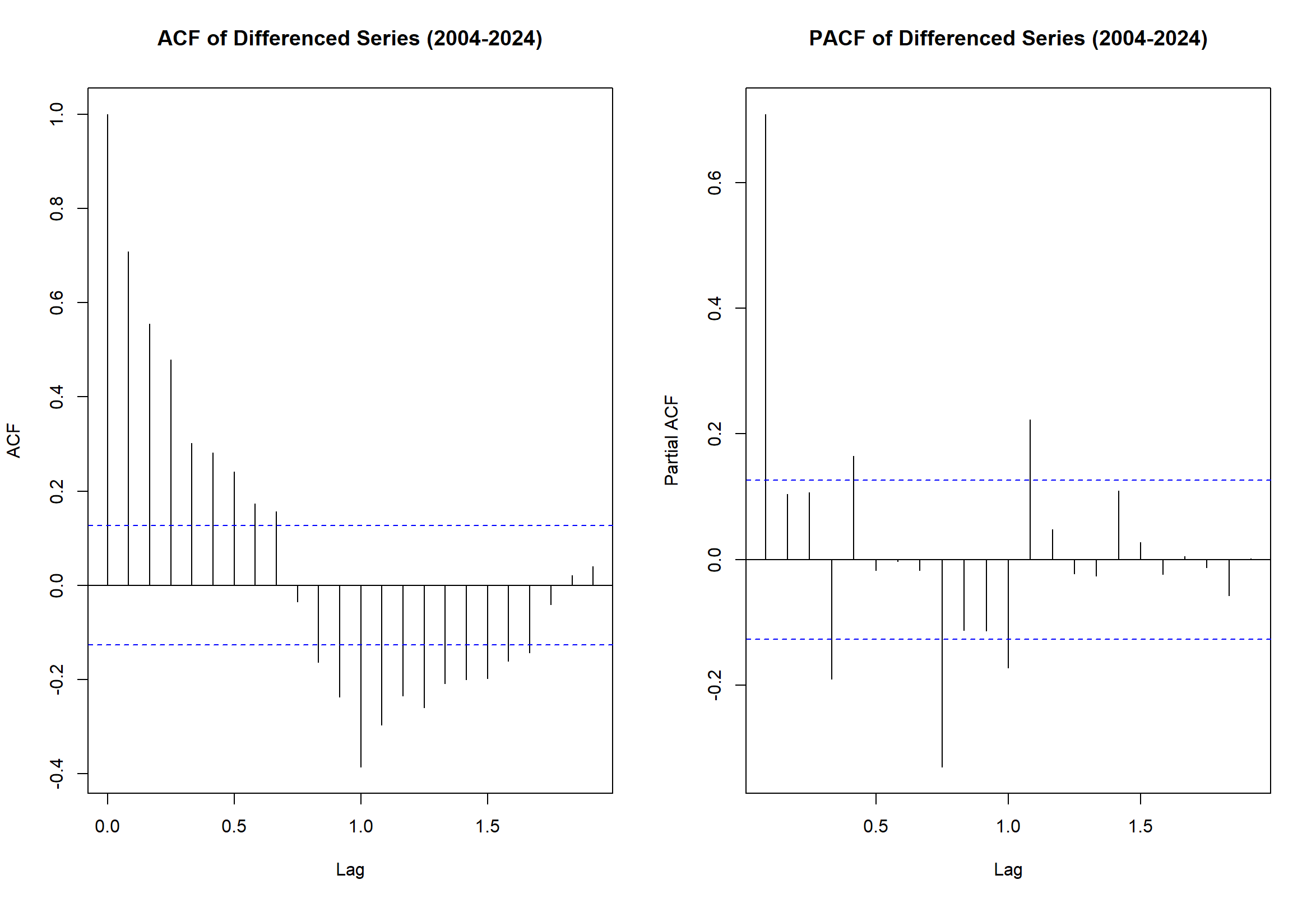


Note: The series was differenced at lag 12 (one seasonal difference) based on the selected SARIMA model ARIMA(3,0,1)(0,1,1)[12] with drift. The autocorrelation function (ACF) shows a negative spike at lag 12 (–0.385), supporting a seasonal MA(1) component. The partial autocorrelation function (PACF) exhibits a significant spike at lag 1 (0.709) and then decays, consistent with a non‑seasonal ARMA(3,1) structure.

**Supplementary Figure S4.** ACF and PACF of the original mortality series, 2004-2024


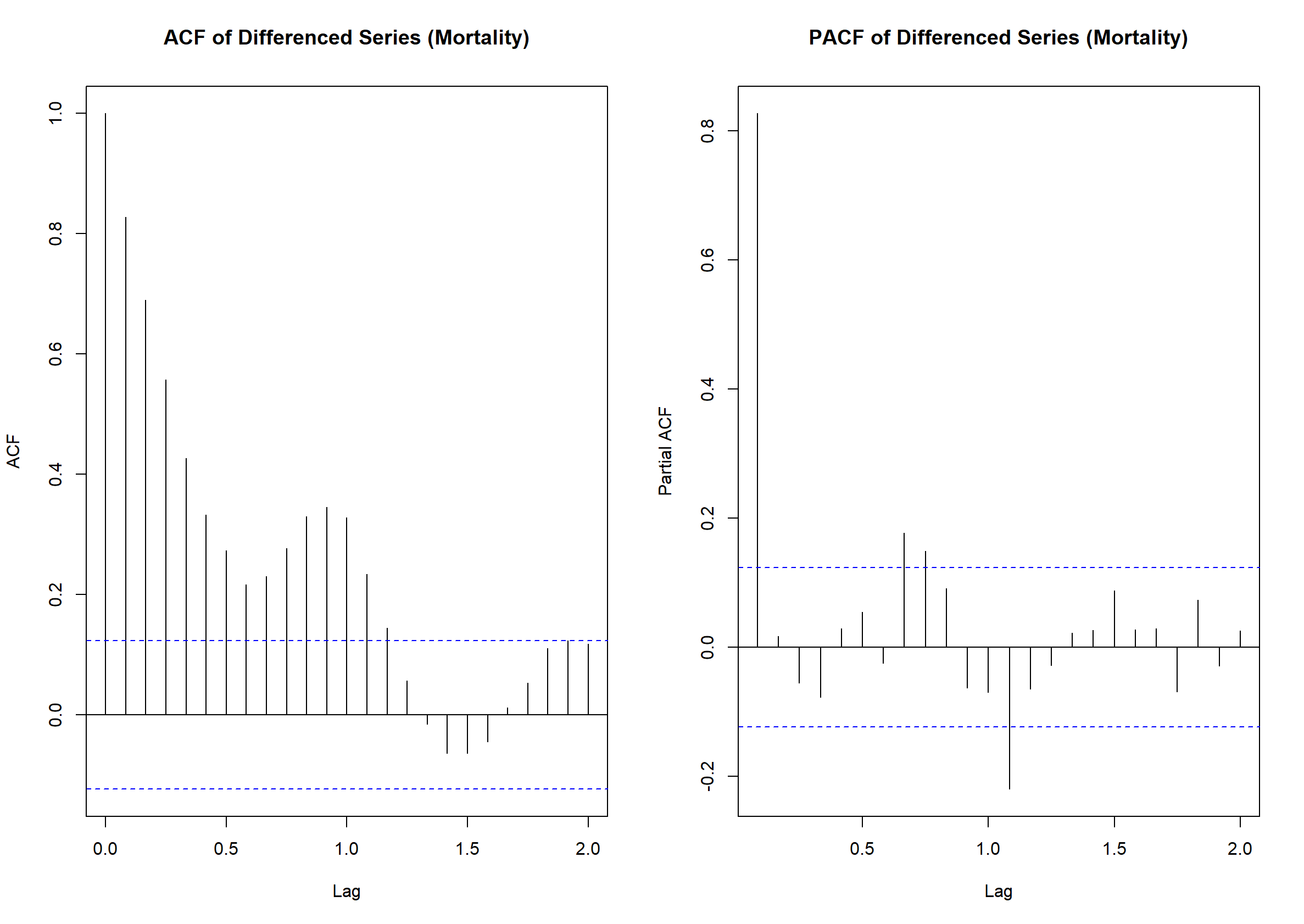


Note: The selected SARIMA model for mortality was ARIMA(1,0,0)(2,0,0)[12] with non‑zero mean (no differencing). The ACF declines gradually and shows a moderate peak at lag 12 (0.3278). The PACF has a strong spike at lag 1 (0.8271) and smaller spikes at lags 12 (-0.0695) and 24 (0.0254), which is compatible with a seasonal AR(2) structure.

**Supplementary Figure S5.** Rolling forecasts vs. actual values for pulmonary tuberculosis incidence in China, 2022-2025


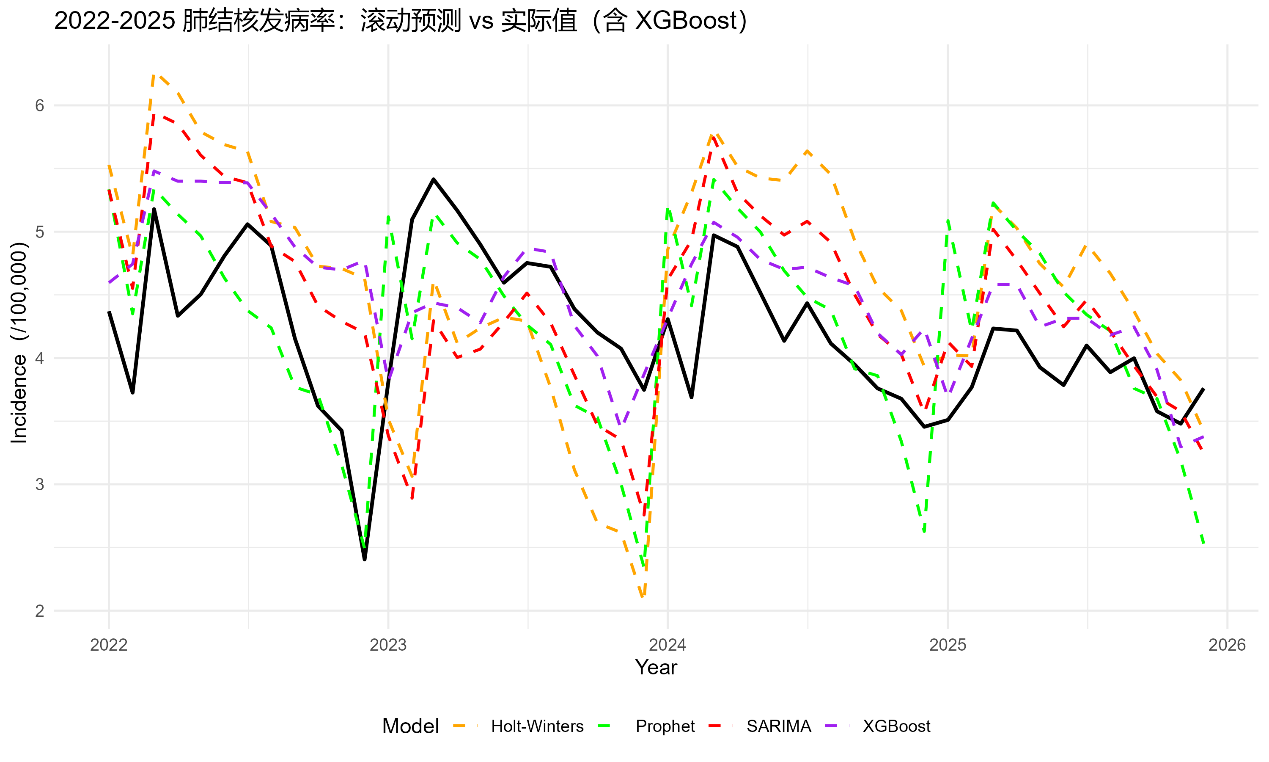


Note: One‑year‑ahead rolling forecasts (dashed lines) from Holt‑Winters, SARIMA, Prophet, and XGBoost models are compared against the observed incidence rates (solid black line). The XGBoost model achieved the lowest forecast errors (MAE = 0.49, MAPE = 12.72%). The rolling‑window scheme used expanding training sets (2004‑2021 → 2004‑2024). Actual test period data are shown for reference.

Legend: Black solid line: Actual observed incidence. Orange dashed line: Holt-Winters model forecasts. Red dashed line: SARIMA model forecasts. Green dashed line: Prophet model forecasts. Purple dashed line: XGBoost model forecasts

**Supplementary Figure S6.** Rolling forecasts vs. actual values for pulmonary tuberculosis mortality in China, 2022-2025


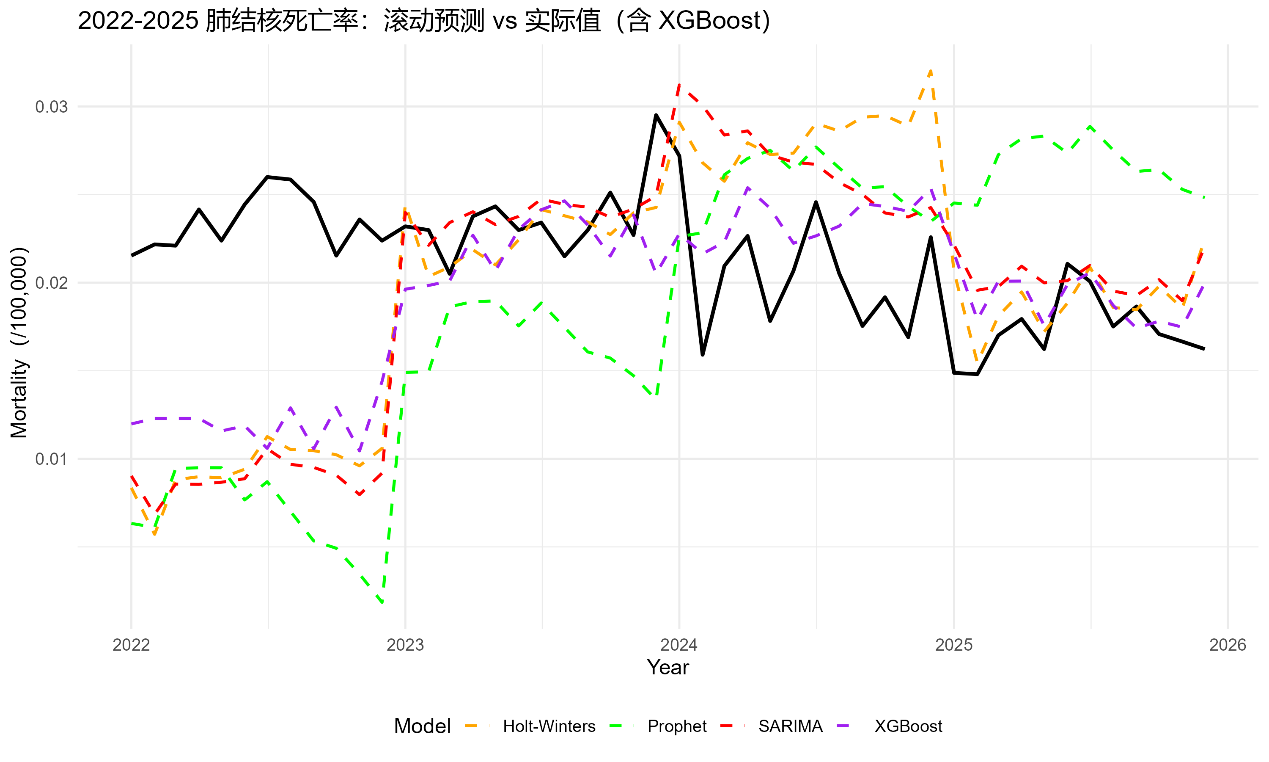


Note: One‑year‑ahead rolling forecasts (dashed lines) from Holt‑Winters, SARIMA, Prophet, and XGBoost models are compared against the observed mortality rates (solid black line). XGBoost significantly outperformed the other models (all p < 0.001, Diebold‑Mariano test), with the lowest MAE (0.0050) and MAPE (23.22%). The rolling‑window scheme was identical to that used for incidence.

Legend: Black solid line: Actual observed incidence. Orange dashed line: Holt-Winters model forecasts. Red dashed line: SARIMA model forecasts. Green dashed line: Prophet model forecasts. Purple dashed line: XGBoost model forecasts.

**Supplementary Table S1.** Comparison of candidate SARIMA models on the last training window, 2004-2024.

| **Model** | **AIC** | **AICc** | **BIC** | **Ljung-Box p (lag=24)** |
| --- | --- | --- | --- | --- |
| ARIMA(3,0,1)(0,1,1)[12] (auto-selected) | 315.74 | 316.22 | 340.10 | 0.1415 |
| ARIMA(3,0,1)(0,1,1)[12] (manual re-fit) | 315.90 | 316.38 | 336.78 | 0.1415 |
| ARIMA(1,0,1)(1,1,1)[12] | 319.85 | 320.33 | 340.73 | **0.0283** |

**Supplementary Table S2**. Monthly forecasts of pulmonary tuberculosis incidence in China, 2026-2030, from the XGBoost quantile regression model (95% prediction intervals).

| **Date** | **Forecast** | **Lower 95% PI** | **Upper 95% PI** |
| --- | --- | --- | --- |
| 2026/1/1 | 3.567141 | 3.75966 | 5.396915 |
| 2026/2/1 | 3.855165 | 3.85724 | 5.46374 |
| 2026/3/1 | 3.814702 | 3.768946 | 5.46374 |
| 2026/4/1 | 3.894212 | 3.691123 | 5.46374 |
| 2026/5/1 | 3.82346 | 3.698228 | 5.466308 |
| 2026/6/1 | 3.976187 | 3.785917 | 5.483995 |
| 2026/7/1 | 4.008168 | 3.669628 | 5.482886 |
| 2026/8/1 | 4.184249 | 3.789372 | 5.495451 |
| 2026/9/1 | 3.975005 | 3.462132 | 5.574164 |
| 2026/10/1 | 3.880112 | 3.388194 | 5.694309 |
| 2026/11/1 | 3.805906 | 3.545921 | 5.743565 |
| 2026/12/1 | 3.878822 | 3.486335 | 5.841983 |
| 2027/1/1 | 3.737938 | 3.653012 | 5.931841 |
| 2027/2/1 | 3.78432 | 3.697694 | 5.960135 |
| 2027/3/1 | 3.731298 | 3.408084 | 5.960135 |
| 2027/4/1 | 3.759914 | 3.450176 | 5.979083 |
| 2027/5/1 | 3.847979 | 3.70917 | 6.00806 |
| 2027/6/1 | 3.788371 | 3.447631 | 6.006728 |
| 2027/7/1 | 3.903591 | 3.793968 | 6.006728 |
| 2027/8/1 | 3.937397 | 3.461298 | 6.006728 |
| 2027/9/1 | 3.955819 | 3.435421 | 6.006728 |
| 2027/10/1 | 3.986776 | 3.225191 | 6.006753 |
| 2027/11/1 | 3.949678 | 3.370049 | 6.006753 |
| 2027/12/1 | 3.929748 | 3.326083 | 6.006753 |
| 2028/1/1 | 3.916647 | 3.526426 | 6.006753 |
| 2028/2/1 | 3.890039 | 3.495747 | 6.006753 |
| 2028/3/1 | 3.817996 | 3.080264 | 6.006753 |
| 2028/4/1 | 3.881339 | 3.037538 | 6.006753 |
| 2028/5/1 | 3.75036 | 3.42679 | 6.006753 |
| 2028/6/1 | 3.780922 | 3.393115 | 6.006753 |
| 2028/7/1 | 3.80529 | 3.512075 | 6.006753 |
| 2028/8/1 | 3.813553 | 3.477467 | 6.006753 |
| 2028/9/1 | 3.911385 | 3.084485 | 6.006753 |
| 2028/10/1 | 3.90871 | 3.496568 | 6.006753 |
| 2028/11/1 | 3.895552 | 3.160282 | 6.006753 |
| 2028/12/1 | 3.912771 | 3.117157 | 6.006753 |
| 2029/1/1 | 4.043028 | 3.44879 | 6.006753 |
| 2029/2/1 | 3.994973 | 3.470375 | 6.006753 |
| 2029/3/1 | 3.929813 | 3.639012 | 6.006753 |
| 2029/4/1 | 3.865473 | 3.657439 | 6.006753 |
| 2029/5/1 | 3.933955 | 3.091541 | 6.006753 |
| 2029/6/1 | 3.920942 | 3.117643 | 6.006753 |
| 2029/7/1 | 3.891323 | 3.413506 | 6.006753 |
| 2029/8/1 | 3.893982 | 3.466596 | 6.006753 |
| 2029/9/1 | 3.861769 | 3.66817 | 6.006753 |
| 2029/10/1 | 3.785958 | 3.47599 | 6.006753 |
| 2029/11/1 | 3.847843 | 3.5941 | 6.006753 |
| 2029/12/1 | 3.972261 | 3.623553 | 6.006753 |
| 2030/1/1 | 3.93184 | 3.07154 | 6.006753 |
| 2030/2/1 | 3.774736 | 3.258763 | 6.006753 |
| 2030/3/1 | 3.902307 | 3.387043 | 6.006753 |
| 2030/4/1 | 3.975129 | 3.441697 | 6.006753 |
| 2030/5/1 | 3.896587 | 3.639012 | 6.006753 |
| 2030/6/1 | 3.867383 | 3.601709 | 6.006753 |
| 2030/7/1 | 3.937953 | 3.145068 | 6.006753 |
| 2030/8/1 | 3.908377 | 3.351972 | 6.006753 |
| 2030/9/1 | 3.86338 | 3.400387 | 6.006753 |
| 2030/10/1 | 3.962986 | 3.065612 | 6.006753 |
| 2030/11/1 | 3.985419 | 3.144724 | 6.006753 |
| 2030/12/1 | 3.800845 | 3.376888 | 6.006753 |

**Supplementary Table S3**. Monthly forecasts of pulmonary tuberculosis mortality in China, 2026-2030, from the XGBoost quantile regression model (95% prediction intervals).

| **Date** | **Forecast** | **Lower 95% PI** | **Upper 95% PI** |
| --- | --- | --- | --- |
| 2026/1/1 | 0.01573 | 0.013736 | 0.021753 |
| 2026/2/1 | 0.014885 | 0.014433 | 0.02184 |
| 2026/3/1 | 0.016497 | 0.014403 | 0.021939 |
| 2026/4/1 | 0.017026 | 0.014598 | 0.020986 |
| 2026/5/1 | 0.017427 | 0.01451 | 0.021967 |
| 2026/6/1 | 0.02007 | 0.014779 | 0.022828 |
| 2026/7/1 | 0.017951 | 0.014909 | 0.022361 |
| 2026/8/1 | 0.017502 | 0.014501 | 0.022672 |
| 2026/9/1 | 0.017615 | 0.014266 | 0.022935 |
| 2026/10/1 | 0.016838 | 0.01402 | 0.023188 |
| 2026/11/1 | 0.016834 | 0.011858 | 0.022511 |
| 2026/12/1 | 0.015912 | 0.01136 | 0.02302 |
| 2027/1/1 | 0.016146 | 0.011279 | 0.025167 |
| 2027/2/1 | 0.015175 | 0.011154 | 0.024825 |
| 2027/3/1 | 0.016437 | 0.011268 | 0.024982 |
| 2027/4/1 | 0.016873 | 0.011268 | 0.023875 |
| 2027/5/1 | 0.017374 | 0.011111 | 0.024933 |
| 2027/6/1 | 0.018631 | 0.011174 | 0.025616 |
| 2027/7/1 | 0.017809 | 0.011175 | 0.024487 |
| 2027/8/1 | 0.017546 | 0.011322 | 0.024794 |
| 2027/9/1 | 0.016909 | 0.011364 | 0.024663 |
| 2027/10/1 | 0.017204 | 0.011364 | 0.025282 |
| 2027/11/1 | 0.016379 | 0.010742 | 0.023728 |
| 2027/12/1 | 0.016983 | 0.010353 | 0.025859 |
| 2028/1/1 | 0.015967 | 0.009517 | 0.026587 |
| 2028/2/1 | 0.016573 | 0.009646 | 0.02627 |
| 2028/3/1 | 0.017059 | 0.009632 | 0.02579 |
| 2028/4/1 | 0.01711 | 0.009612 | 0.025451 |
| 2028/5/1 | 0.017264 | 0.009619 | 0.027442 |
| 2028/6/1 | 0.017531 | 0.009592 | 0.0263 |
| 2028/7/1 | 0.017394 | 0.009053 | 0.026505 |
| 2028/8/1 | 0.017445 | 0.010358 | 0.02607 |
| 2028/9/1 | 0.017427 | 0.011833 | 0.026654 |
| 2028/10/1 | 0.016621 | 0.01064 | 0.024501 |
| 2028/11/1 | 0.01705 | 0.009669 | 0.02505 |
| 2028/12/1 | 0.016462 | 0.009884 | 0.025736 |
| 2029/1/1 | 0.016728 | 0.00852 | 0.026503 |
| 2029/2/1 | 0.017453 | 0.007592 | 0.02697 |
| 2029/3/1 | 0.016527 | 0.00779 | 0.026512 |
| 2029/4/1 | 0.016526 | 0.009181 | 0.026161 |
| 2029/5/1 | 0.017151 | 0.01114 | 0.026743 |
| 2029/6/1 | 0.01741 | 0.008561 | 0.026656 |
| 2029/7/1 | 0.016778 | 0.008902 | 0.026666 |
| 2029/8/1 | 0.016854 | 0.010846 | 0.026442 |
| 2029/9/1 | 0.017067 | 0.012939 | 0.026884 |
| 2029/10/1 | 0.016898 | 0.00993 | 0.026099 |
| 2029/11/1 | 0.016846 | 0.008725 | 0.026632 |
| 2029/12/1 | 0.017268 | 0.009238 | 0.026167 |
| 2030/1/1 | 0.016342 | 0.009669 | 0.026503 |
| 2030/2/1 | 0.016805 | 0.008482 | 0.027284 |
| 2030/3/1 | 0.017309 | 0.00891 | 0.026514 |
| 2030/4/1 | 0.016977 | 0.009199 | 0.026226 |
| 2030/5/1 | 0.016934 | 0.011508 | 0.027277 |
| 2030/6/1 | 0.017322 | 0.009501 | 0.027307 |
| 2030/7/1 | 0.017144 | 0.009121 | 0.027299 |
| 2030/8/1 | 0.017161 | 0.009577 | 0.026514 |
| 2030/9/1 | 0.017128 | 0.011037 | 0.026877 |
| 2030/10/1 | 0.017143 | 0.009286 | 0.026185 |
| 2030/11/1 | 0.017161 | 0.009034 | 0.026745 |
| 2030/12/1 | 0.017073 | 0.008362 | 0.025082 |
